# Time Spent in Nature Is Associated with Increased Pro-Environmental Attitudes and Behaviors

**DOI:** 10.3390/ijerph18147498

**Published:** 2021-07-14

**Authors:** Nicole V. DeVille, Linda Powers Tomasso, Olivia P. Stoddard, Grete E. Wilt, Teresa H. Horton, Kathleen L. Wolf, Eric Brymer, Peter H. Kahn, Peter James

**Affiliations:** 1 Department of Epidemiology, Harvard T. H. Chan School of Public Health, Boston, MA 02115, USA; 2 Channing Division of Network Medicine, Department of Medicine, Brigham and Women’s Hospital and Harvard Medical School, Boston, MA 02115, USA; 3 Department of Environmental Health, Harvard T. H. Chan School of Public Health, Boston, MA 02115, USA; tomasso@hsph.harvard.edu (L.P.T.); gwilt@g.harvard.edu (G.E.W.); pjames@hsph.harvard.edu (P.J.); 4 Population Health Sciences, Harvard University, Boston, MA 02115, USA; 5 Department of Nutrition, Harvard T. H. Chan School of Public Health, Boston, MA 02115, USA; ostoddard@hsph.harvard.edu; 6 Department of Anthropology, Northwestern University, Evanston, IL 60208, USA; thorton@northwestern.edu; 7 School of Environmental and Forest Sciences, University of Washington, Seattle, WA 98195, USA; kwolf@uw.edu (K.L.W.); pkahn@uw.edu (P.H.K.J.); 8 Faculty of Health, Gold Coast Campus, Southern Cross University, Gold Coast 4225, Australia; Eric.Brymer@acap.edu.au; 9 Department of Psychology, University of Washington, Seattle, WA 98195, USA; 10 Department of Population Medicine, Harvard Pilgrim Health Care Institute and Harvard Medical School, Boston, MA 02115, USA

**Keywords:** nature, nature exposure, time in nature, nature affinity, environmental values, pro-environmentalism, environmental education, nature experience, environmental attitudes, environmental behaviors

## Abstract

Urbanization, screen dependency, and the changing nature of childhood and parenting have led to increased time indoors, creating physical and emotional distancing from nature and time spent in natural environments. Substantial evidence from observational and intervention studies indicates that overall time spent in nature leads to increased perceived value for connectedness to nature and, subsequently, greater pro-environmental attitudes and behaviors (PEAB). This narrative review of the recent literature evaluates associations between time spent in nature with values ascribed to nature and nature connectedness, as well as PEAB. We discuss the influence of nature exposure and education in childhood on subsequent development of PEAB in adulthood. We analyze theoretical frameworks applied to this research as well as metrics employed, populations studied, and individual and societal values before presenting limitations of this research. We conclude with suggestions for future research directions based on current knowledge, underscoring the importance of promoting time spent in nature and PEAB in the face of growing challenges to planetary health. Research indicates that overall time spent in nature, regardless of the quality of environmental conditions, leads to increased perceived values ascribed to nature, which is associated with PEAB; however, this literature is predominantly cross-sectional. Furthermore, personal and social factors may influence PEAB. Thus, more longitudinal studies that consider these factors are needed to assess the duration and frequency of time spent in nature in childhood and its impact on PEAB throughout the life course. Identifying contexts which cultivate PEAB and reverse alienation from nature beginning in childhood may better sensitize adults to the urgency of environmental issues such as climate change, which adversely impact individual and environmental health.

## 1. Introduction

There has been a considerable increase in the number of epidemiological studies examining associations between exposure to nature and various health outcomes, primarily in observational research [1,2,3,4]. Generally, exposure to nature, which is defined and measured in different ways across studies and disciplines, demonstrates positive associations with overall physical and psychological health, emotional wellbeing, mortality, and a host of other health outcomes [5,6,7,8,9,10,11]. Studies also indicate that time spent in nature may positively influence pro-environmental attitudes and behaviors (PEAB) through several pathways, including cultivation of biocentric values [12], connection to nature [13], place attachment [14], and psychological restoration [15,16]. Recent studies add empirical evidence that strengthens known associations between childhood nature exposure and time spent in nature and PEAB across cultures [17,18,19,20,21]. However, a growing body of research indicates that children and young adults may be spending less time outdoors than previous generations [22,23,24,25,26], and in doing so, neglect stimulation of major pathways shown to catalyze empathy and care for the environment [27] and different and complex neural pathways developed through mentored introductions to nature [28]. Diminishing exposure to nature may adversely impact pro-environmentalism or individuals’ PEAB, with ensuing negative consequences for the environment [29]. Thus, cultivating PEAB through nature exposure and connection to nature is critical in buffering ecosystem vulnerability in the face of climate change and other environmental stressors of human habitation. Furthermore, PEAB should be examined alongside other health and wellbeing benefits derived from nature contact.

Research on the health benefits of contact with nature and research on environmental sustainability are rarely integrated. Individuals who spend more time in nature tend to be both healthier as well as more disposed toward acknowledging and addressing challenges to planetary health where nature can potentially offer solutions such as the slowing of the climate crisis. Thus, updates to the literature on nature exposure and environmental attitudes/environmental behaviors (EA/EB) should also incorporate environmental sustainability, including climate activity.

Broadly, environmental attitudes are an individual’s beliefs, affect, and behavioral intentions regarding nature and environmentally related activities or issues [30]. Environmental attitudes encompass aspects such as an individual’s environmental reasoning [31,32,33,34], ecological beliefs [35], connection to nature [36], place attachment [37], biophilia [38,39], and willingness to engage in pro-environmental behaviors [17,18,19]. Pro-environmental behavior is defined as environmentally responsible or environmentally protective behavior [20], such as biodiversity conservation [40] or adoption of recycling efforts [41]. Research demonstrates positive associations between environmental attitudes and behaviors [42], with pro-environmental attitudes generally mediating the relationship between nature exposure and pro-environmental behaviors [13,21,43].

Most observational research measures exposure to nature by time spent outdoors or in a natural environment. Nature contact may be intentional, incidental, or indirect, and this contact occurs within diverse cultural, geographic, and ecological contexts [44,45]. Although the benefits of spending time in nature, including urban nature, are well-documented, mounting evidence of population level declines in time in nature may be due to factors including increased urbanization, reliance on technology for work and entertainment, societal changes toward more structured childhood activities, and negative perceptions of nearby nature [22,40,46,47,48,49]. Therefore, interventions to raise awareness of and exposure to nature should be definitionally broad.

Existing reviews of time spent in nature characterize the role of environmental values as forerunners to explicit outcome behaviors, with most dedicated to pro-environmentalism. One comprehensive review evaluated (a) the ways people define nature experiences; (b) current research approaches to investigate EA/EB linkages and interrelationships; and (c) recommendations for future research, including some standardization of criteria for defining and measuring exposure frequency for time in nature as well as a cross-cultural and sociodemographic comparison of effects [21]. Other reviews assess more specific relationships with EB outcomes rooted in more narrow exposure scenarios or interventions, such as those resulting from environmental education initiatives [50]; participation in wildlife recreational activities, which includes environmental stewardship among its many emotional outcomes [51]; and connectedness with nature (CWN) as a predictor of environmentally responsible behavior [52]. A review of theoretical frameworks and intervention strategies to promote EB draws from environmental psychology without explicit reference to time in nature [53]. A final review of the nature–learning relationship examined nature as a facilitator and contextual mediator of cognitive and emotional learning outcomes and environmental stewardship as one learning, though not behavioral, outcome tied to nature contact [54].

In contrast to these, this narrative review incorporates theoretical approaches to cultivating PEAB, paying specific attention to studies which respond to previously highlighted research needs to examine cultural and social variability in PEAB and inequities in nature access and experience which impact PEAB promotion. This narrative literature review briefly examines existing metrics of time spent in outdoor or natural environments during childhood within the fields of environmental health, education, and psychology and introduces definitions and metrics of environmental values, attitudes, and behaviors. We consider conceptual frameworks that connect nature exposure to PEAB. We evaluate evidence for how exposure to nature in childhood influences environmental attitudes and behaviors in adulthood across demographic and cultural contexts, outline research gaps and limitations, and propose future directions to address these gaps.

## 2. Materials and Methods

Narrative reviews consolidate the results of quantitative and qualitative studies that employ diverse methodologies and/or theoretical frameworks, without a strong focus on the statistical significance of the individual study results [48,49]. Our intent to provide a comprehensively broad overview of PEAB research development precluded the use of a strict hypothesis and the narrower article selection required to defend or refute it. We conducted a keyword search-based literature review using PubMed Advanced Search and Web of Science search for studies with titles or abstracts containing “nature exposure,” “time in nature,” “nature affinity,” “environmental values,” “pro-environmentalism,” “environmental education,” “nature experience,” “environmental attitudes,” or “environmental behaviors.” We limited this review to research on human subjects only and included English language-based, international peer-reviewed articles (e.g., primary research, reviews), online reports, electronic books, and press releases. We included both experimental and observational studies and applied a snowballing search methodology using the references cited in the articles identified in the literature search. Each identified item was assessed for relevance by a member of the study team, and we included articles that examined PEAB, as well as how nature exposure is linked to PEAB. This review is not comprehensive but is intended to summarize literature on nature exposure and PEAB. Our search included publications from January 1980, approximately marking the foundational research of significant life experience in nature and environmental education, through June 2021.

## 3. Results

In examining literature on nature exposure and PEAB, we reviewed 67 published articles drawn from multiple disciplines, geographic regions, and study populations. Evidence from the experimental and observational studies presented below represents selected literature from the last four decades on nature exposure and PEAB, primarily from Western countries.

### 3.1. Defining and Measuring Environmental Values

Defining and measuring environmental values has become a major focus of environmental and psychological research as researchers evaluate the origins and drivers of PEAB to design environmental education programs that will promote these attitudes and behaviors.

Van den Bosch and Depledge provide a conceptual framework for understanding how spending time in nature might lead to changes in pro-environmental behaviors [55]. Drawing on evidence from both observational and experimental research, the authors posit that natural environments evoke automatic, unconscious reactions, potentially in the pro-environmental direction, which are mediated by underlying physiological processes [55]. In environmental education research, Brymer and Davids propose an ecological dynamics model of behavior change, which emphasizes interactions between diverse individual, environmental, and task constraints, as a theoretical framework for developing pro-environmental behaviors [56]. In this approach, individuals are considered unique, complex systems comprised of interacting subsystems for thinking, perceiving, learning, and acting. Physical, cultural, social, psychological, and emotional factors can influence these subsystems and influence the adoption of new behaviors. While these frameworks provide broad conceptualizations of how natural environments may affect PEAB, it is important to recognize the diversity of values that shape the way individuals think about and understand their relationship with nature [57].

Consideration of individual and societal values is an integral component in understanding pro-environmental values and behaviors. Defining the value priorities of individuals as a reflection of social experiences and outward behaviors and choices is an important starting point. Schwartz’s Value Theory presents values as desirable goals that serve as guiding principles [58]. The four key features are: (1) A value reflects a belief on the desirability of a certain end state; (2) Values are abstract and transcend specific situations; (3) Values serve as a guiding principle for selecting or evaluating people, behavior, or events; and (4) Values are ordered in a system of value priorities [58,59]. It is important to note that competing values may be activated in different situations, with individualized, value-based choices [60,61] incompatible with cross-cultural or cross-national priorities. These features are useful for environmental research by identifying situations that activate values relevant to pro-environmentalism.

Table 1 outlines three types of values (i.e., intrinsic, instrumental, and relational) mapped against three foci of values related to nature, nature’s contribution to people, and quality of life. Intrinsic values are those inherent to nature and are considered nonanthropocentric. Instrumental values facilitate achieving human ends or satisfying human preferences. Relational values are those that are derived from human relationships with and responsibilities towards nature [62]. Pascual et al. emphasize that values related to nature’s contribution to people are fluid and cannot be placed into a single value category [57]. Both instrumental and relational values can be ascribed to the value of nature’s contribution to people, highlighting that nature’s contributions to people are intertwined with both nature and a good quality of life.

Environmental beliefs, attitudes, norms, intentions, and behaviors are related to the category of self-transcendent values. Self-transcendence is the process of psychological expansion beyond the self [63]. Individuals who favor self-transcendent values are more likely to hold pro-environmental beliefs, compared to those who favor self-enhancement values [30,60,61,64,65,66]. Of particular importance in understanding PEAB are altruistic and biospheric values, two types of self-transcendent values, and hedonic and egoistic values, two types of self-enhancement values [67]. Altruistic values reflect concern with the welfare of others, and biospheric values reflect concern with nature and the environment for the sake of its existence. Hedonic values reflect concern with improving one’s own feelings and pleasure while reducing effort, and egoistic values reflect increasing one’s own resources or power [67]. Individuals who hold altruistic values will likely consider the costs and benefits of their actions to other people. Individuals who hold biospheric values will make choices they believe are likely to benefit the environment [61]. Conversely, those who hold self-enhancement values may consider the personal costs and benefits of environmental actions and act pro-environmentally only when personal benefit outweighs the personal cost (i.e., if the pro-environmental option is cheaper or more comfortable than the more environmentally harmful option) [68]. Consideration of different value systems is integral to future research aimed at understanding how to strengthen the values that promote PEAB.

Measuring environmental values is the first step towards examining patterns of environmental value norms in populations and applying this knowledge of value norms to cultivate PEAB. However, measuring abstract concepts such as environmental values is a challenging task for sociological, psychological, and environmental researchers. A number of environmental value scales exist, including the Environmental-Schwartz Value Survey (E-SVS) [67], Environmental-Portrait Value Questionnaire (E-PVQ) [69], New Environmental Paradigm (NEP) [70,71], Dominant Social Paradigm (DSP) [72], and Ecological World View (EWV) Scale [73] (Table 2). Each of these scales has limitations in methodology and validity and often positions an individual’s values along a linear continuum from anthropocentric (i.e., “anti-environmental”) to biocentric (i.e., “pro-environmental”) worldviews [74]. The two-dimensional measurement of environmental values (2-MEV) expands upon these widely used frameworks and transitions from a unidimensional to two-dimensional framework, which allows investigators to simultaneously explore environmental attitudes and behaviors [75]. Under this approach, environmental values are determined by an individual’s position on two statistically independent dimensions: a biocentric dimension that reflects conservation and protection of the environment (Preservation or P) and an anthropocentric dimension that reflects the utilization of natural resources (Utilization or U) [76]. The framework places individuals into one of four quadrants, a schema that enables mutually high scoring for both Preservation and Utilization, suggesting individuals can simultaneously have biocentric and anthropocentric worldviews [77]. Ad hoc measurements or adaptations of these scales are often created that are best suited to address specific research questions, limiting the generalizability of findings [78].

### 3.2. Nature Experiences and Subsequent Attitudes towards Nature

A large body of literature, primarily in the fields of environmental education, environmental psychology, and environmental tourism, examines formative experiences in nature and how these experiences shape an individual’s later attitudes towards the natural environment (Table 3).

Environmental education research has focused on youth populations who are immersed in educational experiences for short periods of times (e.g., days or weeks), such as National Outdoor Leadership School (NOLS) and environmental-focused summer camps. Researchers examining youth participation in nature-based summer camps versus urban camps found that nature-based camps increased children’s connection to nature (measured via the Emotional Affinity towards Nature scale [79]), pro-environmental attitudes (measured via the New Environmental Paradigm scale [75]), and willingness to display pro-environmental behaviors (pre- and post-test assessment of intention to visit nature and willingness to carry out daily conservation actions and environmental citizenship behaviors) [94]. Researchers have also considered the importance of depth of experience among student populations, comparing deep nature experiences, (e.g., immersive multiday backpacking trips) to more mild nature experiences (e.g., walking along a park trail) [95,96]. Deep nature experiences were associated with more positive attitudes towards nature, even after controlling for inclement weather and negative nature experiences (e.g., mosquito bites).

Research weighing the influence of nature experience in cultivating perspectives sympathetic toward the environment overall tends to assess environmental attitudes in tandem with environmental behaviors. However, individual study results frequently find that expressed pro-environmental attitudes do not easily translate into manifested behaviors, such that EA and EB present as different constructs that require discrete evaluation, often presenting contradictory evidence of the disjoined phenomena. Where one study from New Zealand found that time spent in nature during childhood did not predict PEB or even adult time in natural spaces [80], an industry-based study from Indonesia found attitudes inclined toward environmental protection did in fact predict PEB [81]. Similar discrepancies in study findings have occurred when PEAB were synchronously assessed as separate outcomes: whereas time in nature among UK survey takers elicited both greater appreciation for nature and PEB [82], a longitudinal study of Italian students which evaluated the effects of social identify on EA, EB, and institutional support for environmentalism uncovered negative relationships of EA predicting EB, and vice versa [83]. Such gaps between expressions of and actual engagement in positive environmental change, especially among young adults, are cautionary concerning legislation surrounding climate change, for instance.

Recent reviews investigating associations between exposure to nature and PEAB in children and adults have addressed study gaps brought to light by earlier review papers, such as sparse evidence that environmental education initiatives may lead to long-term environmental behavior change [50] or shortcomings with the design and evaluation of environmental behavioral change interventions [53]. One systematic review and meta-analysis found that adults who are more connected to nature reported greater engagement in pro-environmental behaviors and that a deeper connection to nature may partially explain why some people behave more pro-environmentally than others [84]. Another systematic review of articles published between 2000 and 2016 examined individual psychological, social, and educational outcomes associated with wilderness recreation and found empirical evidence of multiple benefits, including personal development, prosocial behaviors, mental restoration, and environmental stewardship [51]. Additionally, evidence from different disciplines converges around nature’s influence on learning, development, and environmental stewardship [10], with more than 50 studies demonstrating nature’s role in developing PEAB by fostering an emotional connection to nature. An interdisciplinary review of literature from the 1970s to the 1990s using nature connectedness as a core concept emphasized its importance for developing pro-environmental behavioral outcomes such as environmental conservation [52]. However, some inconsistencies exist in the evidence base. One review of 25 studies focusing on the relationship between childhood nature experiences and adult environmental attitudes and behaviors suggested participation in nature-based environmental education programs may lead to short-term but not necessarily long-term improvement in PEAB [85].

The variety inherent in nature exposure is associated with differential PEAB. Different forms of nature contact represented by visits to nature areas, living in a green neighborhood, and nature documentary watching on both nature connectedness and PEAB were studied among nearly 5000 English adults [86]. Weekly visits to nature areas promoted both wellbeing and nature conservation behaviors, though the strength of this relationship varied according to underlying nature connectedness; watching nature conservation programs resulted in significantly higher eudaimonic wellbeing (i.e., related to meaning and purpose) and a greater tendency to engage in pro-environmental behaviors compared to those who did not watch nature documentaries. Individuals who reported higher pre-existing nature connectedness also increased their pro-environmental behaviors after watching conservation documentaries.

An emerging research area focuses on adult perceptions of the environment acquired through environmental tourism intended to minimize the impact on sensitive ecosystems, promote environmental protection, and contribute to local economic sustainability through travel. Research demonstrates that increased environmental tourist participation and nature engagement was associated with increased positive perceptions of nature. Tourist participation suggests that the more individuals learn about and interact with environments, the more topophilic (i.e., place-connected) they feel, with such experiences leading to greater positivity toward nature [87,88].

The existing evidence on nature-related significant life experiences, broadly recognized as influential childhood play outdoors and people who provided healthy introductions to nature, and subsequent PEAB is inconsistent. Associations between significant life experiences in nature and adult PEAB are documented across age, race, and country of origin and point to varied manifestations of PEAB, including environmental knowledge and awareness, climate change concern, and conservation behaviors [89,90,91,92]. There is also evidence of null associations, which nonetheless bring forth other possible relational pathways. An examination of significant life experiences and formative influences outdoors among UK residents involved in climate change education and mitigation concluded that outdoor experiences in childhood were not a significant contributor to adult climate change concern or activism [92]. Rather, social and environmental justice concerns were more important than biospheric concerns, suggesting that climate change concern may be rooted in other relational values and not in nature connectedness. A study of Australian university students showed that positive childhood experiences in nature were associated with pro-environmental attitudes in adults but not necessarily with pro-environmental behaviors [93]. Participants who indicated pro-environmental attitudes did not engage in more pro-environmental behaviors than did participants who held less pro-environmental attitudes.

### 3.3. Socioeconomic Status, Race, Geography, and Access Barriers and Pro-Environmental Attitudes and Behaviors

Different demographic, cultural, and national contexts magnify the challenge of interpreting how exposure to nature influences PEAB. The ways in which socioeconomic status, physical identifiers of race, cultural heritage or place of origin as ethnicity markers, geography, and country of residence that shape and potentially moderate these associations create additional layers of complexity for interpreting early life nature exposure as a predictor of adult PEAB. Inconsistencies in defining nature exposure and interventions across these contexts present a challenge to drawing reliable comparisons. Furthermore, the terminology used to discuss humans’ relationship to nature has an Anglo-centric focus, e.g., stronger individualistic vis à vis community or topophilic identity, likely reflecting many researchers’ origins. One strong exception considers cultural connectedness to the land and environment through the research lens of Indigenous values [97].

Though the scientific literature is sparse, some evidence suggests effect modification of associations between nature exposure and PEAB by culture and regional influence (Table 4). The relatively few existing studies demonstrate how socioeconomic status or race modifies these associations between nature exposure and PEAB. One multinational study conducted in Brazil, the Czech Republic, Germany, India, New Zealand, and Russia supports the cross-cultural generalizability of the relationship between values and attitudes and the structure of environmental concern [30]. Across all countries evaluated, self-transcendent values were positively associated with PEAB, while self-enhancement values were negatively associated with PEAB. Findings from this study are consistent with a separate 14-country multinational study among college students in the U.S., Canada, and several Spanish-speaking countries in the Americas conducted by the same researchers [68]. 

Some studies have explicitly explored race/ethnicity and socioeconomic factors as modifiers of PEAB. For example, researchers examined demographic factors that may influence American college students’ attitudes towards nature and found that white students had fewer negative feelings toward nature (i.e., fear of nature, disconnection with nature) than students of other races [98]. Further, gender, age, parental education, and first-generation college student status were important predictors of attitudes toward nature beyond race. For instance, parent’s educational attainment influenced thinking about both natural and human-made hazards, and first-generation college students were more likely to perceive nature as loathsome and fearful places, which could negatively influence these individuals’ environmental attitudes and behaviors. Historical, cultural, and racial factors play a role in determining attitudes towards nature. For example, an influential study examining African American park visitors’ various outdoor experiences argued that a history of slavery and racial violence has shaped cultural attitudes towards the "great outdoors" and has determined who should and can have access to natural spaces (e.g., national parks) [99]. Research exploring the cultural and historical foundations of continued nonuse of nature [100], the imperatives of nature equity [101], and the need for environmental education shaped by diverse racial perspectives [102] derives from this foundational work. More inclusive assessment studies based on cultural historicity, community values, and urban and rural land uses could elucidate potential modifying roles of race and ethnicity on the relationship between nature exposure and PEAB.

Urbanization has changed patterns in childhood play in nature that may impact adult environmental attitudes and behaviors. An Australian study found that urban dwellers stated they “loved” or “somewhat loved” nature more often than rural dwellers, though expressed love of nature did not differ significantly across socioeconomic classes within urbanicity strata [93]. A study in Kenya showed that economic dependency on surrounding natural environments diminished connectedness with nature and adult PEAB. Specifically, a more traditional lifestyle among the Meru people of Kenya was negatively associated with emotional connectedness to nature [103]. These findings suggest that nature contact under conditions of direct dependence on the natural environment may negatively influence individuals’ feelings toward nature. 

Barriers to positive nature experiences are not primarily weather based, although certain types of weather (e.g., rain, cold temperatures) restrict people from spending time in nature [51]. In addition, research suggests that prior perceptions of weather and personal preferences play a role in nature experience [52]. For instance, people from colder climates are more likely to spend time in nature, regardless of degree of cold, whereas people from warmer climates are more likely to perceive cold temperature as a barrier to engaging in nature experiences. Furthermore, more time spent in nature is associated with increased self-worth and self-efficacy in the face of challenges [84]. Studies of environmental experience across different natural landscapes (i.e., varying vegetation types and densities) reveal that isolated and enclosed spaces appear to be less desirable than forested areas and open landscapes or viewsheds, suggesting that natural landscape type might moderate experiences in nature [85,104].

## 4. Discussion

Research indicates that overall time spent in nature, regardless of the quality of environmental conditions, leads to increased perceived values ascribed to nature, which is associated with PEAB [105]. While associations between time spent outdoors or in nature in childhood and PEAB is well-studied, research exploring the influence of time spent outdoors in nature and demonstration of PEAB is predominantly cross-sectional and not assessed longitudinally. Furthermore, a multitude of personal and social factors, including childhood experience, knowledge and education, personality, values, worldviews, goals, felt responsibility, cognitive biases, place attachment, age, gender, religion, urban–rural differences, norms, socioeconomic status, and proximity to problematic environmental sites, may influence PEAB [106,107]. Thus, more longitudinal studies that consider these factors are needed to assess duration and frequency of time spent in nature in childhood and its impact on PEAB throughout the life course.

There are several explanations for the shallow evidence base across demographic and cultural contexts analyzing childhood predictors of adult environmental attitudes and behaviors. First, the current evidence base is limited by the use of convenience samples of university students, which minimizes variability in participants’ socioeconomic status, and is conducted in relatively homogeneous societies (e.g., Scandinavia, Australia) rather than across subpopulations of interest. Second, comparisons of adult environmental attitudes and behavioral outcomes across demographics or countries require that time spent in nature be consistently defined at baseline in a way that ensures inclusivity and equity. Third, cross-cultural comparisons should consider the quantity and quality of the surrounding biodiversity that may impact associations with nature exposure and urban socioeconomic factors. Furthermore, inconsistent development and deployment of tools to measure nature exposure make it difficult to identify which specific elements of nature contact most directly stimulate pro-environmental attitudes and behavioral change.

### 4.1. Data Gaps and Limitations

While the literature demonstrates associations between exposure to nature and PEAB, much is still unknown, such as how different nature qualities and interactions with nature differentially impact individuals’ PEAB [44,108], how to account for different types of nature exposure (e.g., green space, blue space, deserts) [109], and the mechanistic pathways for the associations of nature contact and PEAB.

Several limitations constrain the research of how nature experiences shape PEAB. A lack of longitudinal studies limits knowledge on how perceptions of nature change over time within individuals and how singular events (e.g., environmental tourism and short-term educational experiences in youth) may impact one’s long-term perception of nature. Many of the studies we examined are cross-sectional, making it difficult to determine whether positive associations between nature experiences and PEAB attenuate with time elapsed since the experience. Additional research examining mechanistic pathways activated by childhood nature exposure and outdoor play that may foster PEAB throughout the life course is needed. 

It is critical to recognize that what qualifies as a significant nature experience can vary across individuals and populations, which may result in differing outcomes related to connection to nature in childhood. Furthermore, nature affinity in childhood may evolve over time as one’s experiences (e.g., frequency of experience, type of experience) in nature change [110]. Current findings from small-scale interventions evaluating nature exposure and changes in environmental attitudes and behaviors may not translate to larger system-wide studies to increase nature exposure across population groups.

It remains unclear how differences in frequency and the duration of nature exposure across the life course in understudied populations might affect PEAB. While research indicates that aging positively impacts perceptions of nature [111,112], too few studies have considered the influence of disabilities [113]; older and disabled individuals encounter more barriers to spending time in and making connections with the natural world [114]. Most research to date is focused on higher-income, Western, and relatively homogeneous populations, which limits opportunities to compare the influence of nature exposure both within and across populations by urban–rural gradient, sociodemographic characteristics, ecological knowledge, cultural attributes, and prioritized values. Socioeconomic status may pose a significant barrier to engaging in formative childhood nature experiences which are associated with the connection to nature in adulthood [115]; however, few studies have examined financial means and access to nature experiences [116,117].

### 4.2. Future Research Directions

#### 4.2.1. Enhancing Study Design

Availability of and access to nature is important but sometimes insufficient to encourage its use, which may play a role in the development of PEAB. Identifying interventions and system-level changes that can lead to increased and more equitable exposure to and engagement with nature is necessary. Few longitudinal studies exist that track whether, how, and why individuals have contact with nature and how their attitudes and behaviors towards nature evolve over longer time periods. Consequently, many researchers call for high-quality longitudinal, population-level analyses to build upon the plethora of evidence from small-scale nature interventions. It may be helpful to describe nature in terms of availability, access, quality, and usage to examine associations across socioeconomic status, age, race, geographic location, and cultures to ensure inclusivity. Although some longitudinal research has been conducted [118,119], more is needed at the population level that integrates previously mentioned factors (e.g., socioeconomic status, age, race).

A substantial amount of research documents associations between children’s exposure to nature and various emotional and cognitive outcomes and the development of pro-environmental attitudes and behaviors. Moving forward, there is a need for multinational studies featuring multiple sites of varying population densities, socioeconomic levels, and demographic makeups that will assess the frequency and duration of nature exposure and subsequent attitudes towards nature among a cohort of children. Relating these predictor variables to standardized outcomes (e.g., PEAB) will aid in the formulation of a baseline set of global indicators that may also help delineate patterns of frequency and duration in nature and outcomes predicted by measured contact. Another important area of interest is the role of nature exposure on children in transition, such as children of recent immigrants, children whose families have recently urbanized, or children in shelters or temporary homes. Determining how these factors might impact PEAB in both childhood and adulthood is timely and critical.

#### 4.2.2. Improving Assessment of Pro-Environmental Attitudes and Behaviors

Future research on environmental values should focus on revising measurement tools to encompass research needs and determine which specific types of nature contact most directly impact pro-environmental behaviors to inform development of environmental interventions and programs. Consideration of different values is integral to future research aimed at understanding ways to prioritize PEAB. Future studies should aim to determine how biospheric values can be strengthened and activated to increase pro-environmental behaviors [61]. While some two dozen iterations of a nature connectedness scale have been developed to assess the predictive relationship of connection to nature and PEAB, relatively few studies have looked at environmental behaviors as an outcome of nature exposure [86]. More research on the linkages between PEAB with nature and global sustainability outcomes is warranted. Individuals who spend more time in nature tend to be both healthier as well as more inclined towards PEAB to mitigate climate change. Thus, research into nature exposure and environmental attitudes and behaviors should be updated to include climate-related outcomes.

#### 4.2.3. Furthering Mechanistic Research

The mechanisms through which early time spent in nature might influence PEAB are not well understood. Early exposure to nature could be based on positive, significant life experiences or, conversely, exposure could serve as a buffer against the later-life effects of adverse childhood experiences that influence PEAB [120]. One potential mechanistic pathway for PEAB is attention restoration, with perceived restorativeness from nature reinforcing the relationship between environmental knowledge and pro-environmental behavior [121]. A second proposed mechanism is detachment from materialistic values, where one imagines personal worth derived from possessions and status, in spite of the negative environmental impact of overconsumption. Exposure to nature has been associated with decreased materialistic impulses among individuals directed to nature-based imagery relative to viewers of urban scenes and activated altruism as a mediator of diminished materialism [122], lending credence to this mechanism. 

Disentangling mechanistic pathways will be critical for designing and evaluating interventions that aim to promote PEAB, such as environmental education programming [106]. The field would benefit immensely from studies that assess PEAB beginning early in life and following participants throughout the life course to examine changes to individual set (e.g., cultural values) and setting (e.g., built and natural environments). Future mechanistic studies should include ecological momentary assessments, which involve the repeated sampling of participants’ current behaviors, emotions, and experiences in real time as participants move throughout natural environments [123], and the effect of in situ nature contact on pro-environmental inclinations. The theories presented in this section are anthropocentric, and the field would benefit immensely from future research using a relational theoretical approach, where humans are considered part of, rather than distinct from, ecology [124,125].

## 5. Conclusions

Substantial evidence from observational and intervention studies indicates that overall time spent in nature is associated with increased perceived value for and connection to nature and, subsequently, greater PEAB. The current evidence base is limited by several factors, including primarily cross-sectional studies, a lack of mechanistic research, and inconsistencies in the assessment of nature exposure and PEAB. We suggest several future research directions (enhancing study design, improving assessment of PEAB, and furthering mechanistic research), each of which underscores the importance of promoting time spent in nature and PEAB in the face of growing challenges to planetary health.

## Figures and Tables

**Table 1:** Diverse values related to nature, nature’s contributions to people, and good quality of life.

Foci of Value	Type of Value	Examples
Nature	Intrinsic (non-anthropocentric)	Animal welfare/rights
		Gaia, Mother Earth
		Evolutionary and ecological processes
		Genetic diversity, species diversity
Nature’s Contributions to People	Instrumental (anthropocentric)	Habitat creation and maintenance, ecosystem services, pollination and propagule dispersal, regulation of climate
		Food and feed, energy, materials
Good Quality of Life	Relational (anthropocentric)	Physical and experiential interactions with nature, symbolic meaning, inspiration
		Physical, mental, emotional health
		Way of life
		Cultural identity, sense of place
		Social cohesion

Note: Adapted from Pascual et al., 2017 [57].

**Table 2:** Brief descriptions of selected environmental value scales.

Environmental Value Scale	Citation	Scale Description
Environmental-Schwartz Value Survey (E-SVS)	Steg et al., 2014 [67]	A 16-item scale containing descriptions of the biospheric, altruistic, hedonic, and egoistic values. Items are assessed on a 9-point scale (ranging from “−1 opposed to my values” to “0 not important” to “3 important” to “6 very important” to “7 of supreme importance”) indicating how important each value is as a guiding principle in life.
Environmental-Portrait Value Questionnaire (E-PVQ)	Bouman et al., 2018 [69]	A 17-item scale adapted from E-SVS. Participants asked to respond on a 7-point scale (ranging from “1 not like me at all” to “7 very much like me”) how much another (gender-matched) person is similar to themselves in terms of biospheric, altruistic, hedonic, and egoistic values.
New Environmental Paradigm (NEP)	Arcury et al., 1986 [70]; Dunlap and Van Liere 2008 [71]	A 12-item scale measuring acceptance of the NEP, which includes questions related to emerging environmental issues (e.g., limits to growth, balance of nature, anti-anthropocentrism). Items are assessed on a 4-point Likert scale and summed to give a rating scale ranging from 12 (i.e., complete rejection of the NEP) to 48 (i.e., complete acceptance of the NEP).
Dominant Social Paradigm (DSP)	Dunlap and Van Liere 1984 [72]	A 37-item scale measuring commitment to society’s dominant values and beliefs across eight dimensions (e.g., support for laissez faire government, support for status quo, support for private property rights, faith in science and technology, support for individual rights, support for economic growth, faith in material abundance, faith in future prosperity). A general negative association between DSP and environmental concern is strongly supported in the literature.
Ecological World View (EWV) Scale	Blaikie 1993 [73]	A 24-item scale, including original and modified items from the NEP (6), DSP (6), and Richmond and Baumgart (8) scales. Items are assessed on a 5-point Likert scale (ranging from “strongly agree” to “strongly disagree”) and summed to create a score assigned to one of the following EWV categories: Very High, High, Moderate, Low.
Two-Dimensional Measurement of Environmental Values (2-MEV)	Bogner 2018 [75]	A 21-item scale measuring factors of preservation, utilization, and appreciation. Items are assessed on a 5-point Likert scale (ranging from “1 I totally disagree” to “5 I totally agree”). A nature-oriented person would score high in preservation and appreciation, but low in utilization.

**Table 3:** Studies associating forms of nature exposure and experiences with PEAB outcomes.

Nature Experience	Citation	Direction of PEAB Outcome
Short-term nature immersion, e.g., NOLS, summer camp	Müller et al., 2009 [79]	Nature-based camps vs. urban camps increased children’s connection to nature, PEA, and willingness toward PEB engagement. Deeper nature experiences led to more positive attitudes toward nature vis à vis mild experiences.
PEA vs. PEB discretely assessed	van Heezik 2021 [80]	Childhood time in nature not associated with increased PEB or adult time spent in nature in N.Z. study.
	Adam 2021 [81]	Industrial study of pro-EA and education led to increased PEB in Indonesia.
PEAB assessed in tandem	Alcock 2020 [82]	Time in nature increased nature appreciation in PB in large U.K. study.
	Prati 2017 [83]	The influence of social identity among Italian college students on separately measured EA/EB and institutional support for the environment showed negative relationships for EA on EB and for EB on EA.
Review papers of nature exposure and PEAB	Gralton et al., 2004 [50]	Sparse evidence that environmental education leads to long-term EB change.
	Steg and Vlek 2009 [53]	Found the design and evaluation of EB change interventions inconsistent.
	Whitburn et al., 2019 [84]	Meta-analysis found that adults more connected to nature appeared more engaged in PEB, an effect partially explained by deeper nature connection.
Review paper of wilderness recreation experience	Holland et al., 2018 [51]	Wilderness-based recreation led to enhanced personal development, pro-social behaviors, mental restoration, and environmental stewardship outcomes.
Review of childhood nature experiences	Bratman et al., 2019 [10]	Childhood nature experiences vital for learning, development, and nurturing environmental stewardship.
	Zylstra et al., 2014 [52]	Nature connectedness essential for cultivating PEAB in this multidisciplinary review.
	Wells and Lekies 2016 [85]	Nature exposure and environmental education in childhood associated with short-term but not long-term PEAB.
Varied forms of nature exposure	Martin et al., 2020 [86]	Variability of nature exposure associated differently with PEAB. Nature visits, living in green neighborhoods, and viewing nature documentaries induced different strengths of PEAB and wellbeing, as also influenced by levels of nature connectedness.
Environmental tourism	Lee and Jan 2015 [87] Su, Huang and Pearce 2018 [88]	Tourists exposed to nature through learning and education more positively perceived nature-based environments.
Nature-related significant life experiences	D’Amore and Chawla 2012 [89] Stevenson et al., 2014 [90] Hsu 2009 [91]	SLE associated with increases in environmental knowledge and awareness, climate change concern, and conservation behaviors.
	Howell and Allen 2019 [92]	Outdoor experiences in childhood shown not to contribute to adult climate change activism in U.K., and social/environmental justice concerns outweighed biospheric concerns.
	Broom 2017 [93]	Those expressing PEA were not more prone to PEB than those not espousing PEA.

**Table 4:** Studies associating specific cultural and sociodemographic attributes with PEAB outcomes.

Cultural or SD Attribute	Citation	Direction of PEAB Outcome
Nationally measured cultural values toward the environment	Schultz et al., 2005 [30] Schultz and Zelezny 1999 [68]	Multinational studies support cross-cultural generalizability relating EA and environmental concern. Self-transcendent values link to PEAB. Same outcomes realized across the Americas.
Race, ethnicity, and SES as PEAB modifiers	Taylor 2019 [98]	Race but also academic interests, gender, age, parents’ education, and first-generation college status impact how college students think of nature.
	Finney 2014 [99]	NPS origins of 1964 Wilderness Act reflect prevailing cultural leadership of time and not current diversity of user public.
	Theriault and Mowatt 2020 [100]	History of African American relationship toward wilderness is examined through dialectic of historical periods and environmental affordances.
	Jelks et al., 2021 [101]	Review of green gentrification of marginalized communities suggests a worsening divide in health and wellbeing between old and new residents.
	Stapleton 2020 [102]	Sociohistorical shaping of race-based perspectives toward environmental issues requires that environmental education be recast to reflect broader experience.
Urbanization patterns	Broom 2017 [93]	Positive childhood exposures to nature resulted in increased EA but did not necessarily translate into EB.
	Marczak and Sorokowski 2018 [103]	Despite general pro-nature attitudes among Kenyan agriculturalists, economic dependency on natural environments associated negatively with nature connectedness.
Barriers to accessing positive nature experiences	Holland et al., 2018 [51]	Examines unique contributions of wildlife experience, finding value in programmatic and leadership aspects, and research gaps on setting’s importance.
	Zylstra et al., 2014 [52]	Measured connectedness with nature manifests as prerequisite for PEAB and socially desired conservation outcomes.
	Whitburn et al., 2019 [84]	Meta-analysis of nature connectedness finds significant and positive relationship with PEAB.
	Lekies and Wells 2016 [85] Kellert 2018 [104]	Childhood experiences in nature associated with adult environmentalism, with wild nature experiences more strongly manifested than with domesticated nature.

## Data Availability

No new data were created or analyzed in this study. Data sharing is not applicable to this article.
